# COVID-19: Impact on linguistic and genetic isolates of India

**DOI:** 10.1038/s41435-021-00150-8

**Published:** 2021-10-11

**Authors:** Prajjval Pratap Singh, Prashanth Suravajhala, Chandana Basu Mallick, Rakesh Tamang, Ashutosh Kumar Rai, Pratheusa Machha, Royana Singh, Abhishek Pathak, Vijay Nath Mishra, Pankaj Shrivastava, Keshav K. Singh, Kumarasamy Thangaraj, Gyaneshwer Chaubey

**Affiliations:** 1 Cytogenetics Laboratory, Department of Zoology, Banaras Hindu University, Varanasi, 221005 India; 2 Department of Biotechnology and Bioinformatics, Birla Institute of Scientific Research Statue Circle, Jaipur, Rajasthan India; 3 Amrita School of Biotechnology, Amrita University Kerala India, Vallikavu, 690525 India; 4 Centre for Genetic Disorders, Institute of Science, Banaras Hindu University, Varanasi, 221005 India; 5 Department of Zoology, University of Calcutta, Kolkata, 700019 India; 6 Department of Biochemistry, College of Medicine, Imam Abdulrahman Bin Faisal University, Dammam, Saudi Arabia; 7 CSIR-Centre for Cellular and Molecular Biology, Hyderabad, 500007 India; 8 Academy of Scientific and Innovative Research, (AcSIR), Ghaziabad, 201002 India; 9 Department of Anatomy, Institute of Medical Sciences, Banaras Hindu University, Varanasi, 221005 India; 10 Department of Neurology, Institute of Medical Sciences, Banaras Hindu University, Varanasi, 221005 India; 11 Department of Home (Police), DNA Fingerprinting Unit, State Forensic Science Laboratory, Government of MP, Sagar, India; 12 Department of Genetics, School of Medicine, University of Alabama at Birmingham, Kaul Genetics Building, Birmingham, AL USA; 13 Centre for DNA Fingerprinting and Diagnostics (CDFD), Hyderabad, 500039 India

**Keywords:** Structural variation, Viral infection

## Abstract

The rapid expansion of coronavirus SARS-CoV-2 has impacted various ethnic groups all over the world. The burden of infectious diseases including COVID-19 are generally reported to be higher for the Indigenous people. The historical knowledge have also suggested that the indigenous populations suffer more than the general populations in the pandemic. Recently, it has been reported that the indigenous groups of Brazil have been massively affected by COVID-19. Series of studies have shown that many of the indigenous communities reached at the verge of extinction due to this pandemic. Importantly, South Asia also has several indigenous and smaller communities, that are living in isolation. Till date, despite the two consecutive waves in India, there is no report on the impact of COVID-19 for indigenous tribes. Since smaller populations experiencing drift may have greater risk of such pandemic, we have analysed Runs of Homozygosity (ROH) among South Asian populations and identified several populations with longer homozygous segments. The longer runs of homozygosity at certain genomic regions may increases the susceptibility for COVID-19. Thus, we suggest extreme careful management of this pandemic among isolated populations of South Asia.

## Introduction

It has been more than 18 months since the first case of COVID-19 was reported in India. Till now, India has been hit by two major waves with several hundred thousand death toll [1]. The devastating second wave was mainly driven by the alpha and delta variants [2, 3]. Researches on the delta variant have shown that it is more than twice as contagious as the Wuhan strain [4, 5]. Before the second wave in India, the third sero-survey conducted during months of December-January, has reported only 21.4% seropositivity [6]. Nevertheless, by the time the second wave arrived, a large number of seropositive people had exhausted the antibodies [7]. Perhaps, this gave an open field to alpha and delta virus variants to sweep. Otherwise, both of these variants were reported in India by the end of 2020, but their ruthless form was seen from April 2021 onwards [8]. This indicates that the waning antibody was the key driving force behind the second wave [7, 9], while the alpha and delta virus variants catalysed the intensity [3, 5, 8].

In the fourth serosurvey conducted by ICMR (Indian Council of Medical Research) during June-July 2021, the seropositivity have been found among 68% of the people [10], which is more than three times larger to third serosurvey [6]. The presence of antibodies in large number of people reflect the predominance of the second wave. The seropositivity in such a large population also suggests that a third major nationwide outbreak is unlikely in the recent past. The COVID-19 cases has been reported each and every regions of India, however, it is not known that how it has impacted the isolated and smaller populations [11].

With the global range expansion of coronavirus SARS-CoV-2, it is a matter of concern to protect vulnerable tribal populations from contagion. Various reports from Brazil have suggested that many of the indigenous communities were hard hit by the coronavirus SARS-CoV-2 [12–16]. India is a country of diverse endogamous tribal populations, speaking various languages [17]. Altogether, tribal populations make 8% of the total Indian census with some of major tribals e.g., Gond, Kol and Bhil populations, who are millions in number [18]. Yet, many of the South Asian tribal populations have experienced severe bottlenecks and are less than a thousand in numbers [19]. There has not been any study so far, on the impact of COVID-19 among these isolated and smaller populations.

In a broader demographic perspective, South Asia is a diverse place with hundreds of ethnolinguistic groups [20]. This is due to long term isolation, genetic drift and endogamy which collectively created unique genetic profile of South Asians [21]. Generally, high level of genetic diversity for a population implies high heterozygosity [22, 23]. This high level of genetic diversity is beneficial to populations for several reasons. In this context, when the genes of individuals in a population vary greatly, it facilitates the populations for better fitness, including survival against infectious diseases [24, 25]. Thus in case of pandemics, the greater diversity in a population reduces the risk of extinction. The COVID-19 low case fatality rate among South Asians was likely due to multiple factors including genetics [26–28] and prior exposure to various pathogens [29, 30]. However, it is important to note that the East Asian-specific signal of positive selection against coronavirus has not been observed among South Asian populations [31].

We inherit every single copy of chromosomes from each of our parents. Our genome contains several homozygous segments or haplotypes where we receive identical or different copies from each of our parents. In consanguineous marriages, the chances of receiving identical copies are high [32]. These identical copies are also known as Runs of Homozygosity (ROH) [33, 34]. The genetic drift for a smaller population tends to increase the ROH. Studying ROH is important for understanding underlying levels of genetic variation [35]. ROH has been used extensively to study population structure, demographic history and genetic structure of complex diseases [33, 34]. It has been shown that the populations with longer ROH are enriched for deleterious variations [35–38]. Though, most of the South Asian populations carry a high level of genetic diversity, few genetic and linguistic isolates as well as historically migrated populations may have low effective population sizes (Ne) and experienced bottleneck and drift in the past. Hence, longer ROH carrying populations may have greater risk to ongoing pandemic. Here, we have studied the Runs of Homozygosity (ROH) among South Asian populations and found out that many of the smaller and isolated populations have high number of long ROH segments.

## Materials and methods

We have used publicly available datasets on Indian populations to estimate the Runs of Homozygosity (ROH) [39, 40]. PLINK 1.9 [41], was used for data management. The ROH for each of the populations was calculated using PLINK 1.9 [41]. We have used '--*homozyg'* function to perform the analysis. For the calculations, we have used 1000 kb windows size with a minimum of 100 SNPs per window allowing one heterozygous and five missing calls per window. The designated window sequentially scans each and every individual and estimate for proportion in a homozygous window for every SNP.

## Results and discussion

**Fig. 1:**
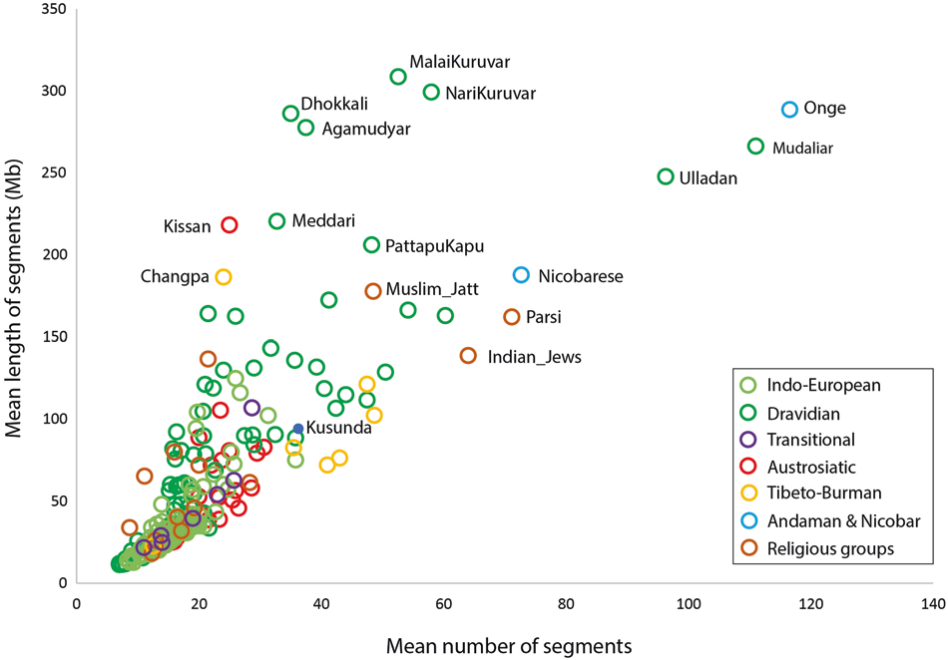
Two dimensional plot of mean number (X-axis) vs mean length (Mb) (Y-axis) of homozygous chromosomal segments among various South Asian populations. Populations with higher than 150 Mb chromosomal segments as well as linguistic isolates have been marked.

Among South Asian ethnic groups, majority of the populations have smaller and fewer number of ROH segments, whereas few of isolated as well as historically migrated populations carried ROH of longer and larger in size (Fig. 1). Historically migrated populations such as Parsis and Jews have their unique demographic history with smaller numbers of past effective populations sizes (Ne) and follow strict endogamy [40, 42, 43]. These groups have migrated to South Asia in the last two millennia with limited founders. The molecular data further revealed that there was a sex-biased admixture with the local females followed by a high level of endogamy [40, 42, 43]. With their low level of heterozygosity these historically migrated populations may have a higher risk of COVID-19.

Among the studied groups, Andaman Islanders have the highest number as well as longest ROH segments (Fig. 1). Great Andamanese (census 43), Onge (census 100), Jarawa (census 375) and Sentinels (census 39) are the aboriginal tribal populations of these islands. Genetic studies on them (the genetic study of Sentinels have not been done yet), have suggested their deep rooted ancestry sharing with the South Asian, East Asian, Southeast Asian and Papuan populations [44–47]. It has been shown that much of the East and Southeast Asian populations are derived from the admixture of Andaman and Tianyuan [48] related ancestries [49]. Andaman Islanders live in protected areas and the general public is not allowed to interact with them. However, seeing some of the past experiences [50] and number of cases at the Island among the general population, they are at greater risk, mainly from illegal intruders and health workers.

Studies have identified *ACE2* as a host receptor for the SARS-CoV-2 [51, 52]. It has been shown that a polymorphism rs2285666 (G > A)of ACE2 gene of X chromosome may increase the expression level upto 50% [53–55]. This polymorphism was also widespread in South Asia and the haplotype associated with this SNP was shared with the East Eurasian populations [28]. Another SNP rs10490770 (T > C) at chromosome 3, introgressed from Neanderthal was also found to be associated with the disease severity mainly among European populations [56]. We have examined both of the SNPs with the Indian statewise infection and case fatality rates, and found a significant association of rs2285666 (but not for rs10490770) [57]. Interestingly, SNP rs2285666 (A) showed a clinal distribution with East and West Eurasia, whereas SNP 10490770 (C) had a frequent distribution primarily in the South Asia [57].

Looking at the clinal distribution of the SNP rs2285666, one may also argue its arrival to South Asia from East and Southeast Asia via geneflow [58, 59], and an isolation by distance (IBD) model for its present distribution. We agree that the spatial distribution of this SNP is significantly associated with the East/Southeast Asian -specific ancestry (*R*^2^ = 0.76; *p* = 9.44 × 10^−6^). Nevertheless, in comparison with the limited language associated (Austroasiatic and Tibeto-Burman), spatial distribution of East/Southeast Asian-specific ancestries [59–61], this SNP is much more frequent and widespread, well beyond the linguistic boundaries in South Asia [27, 57]. Moreover, a recent study on the hospital samples have reported a twofold increase for infection risk as well as threefold more chance of mortality with the risk allele rs2285666 (G) polymorphism [62].

In order to understand the susceptibility of isolated Andaman Islanders, we have estimated frequency of these SNPs among Jarawa and Onge populations. Notably, despite their closer genetic affinity with the ancestral East/Southeast Asian populations [45, 49], they have high frequency of risk allele (C) of rs10490770 (Jarawa 0.26 and Onge 0.29). Such high frequency of Neanderthal-specific allele adds an interesting aspect keeping in mind the 25KYA (Kilo Years Ago) split time with the South Asian populations [63]. For the *ACE2* risk polymorphism rs2285666 (G), the Jarawa and Onge showed frequency of 0.58 and 0.35, respectively. If we compare Tibeto-Burman or Austroasiatic populations (with relatively smaller ROH segments) analysed in the present study, they always tend to show significantly (two tailed *p* < 0.001), lower frequency of the risk alleles for SNP rs2285666 (G) (Table 1). Thus, here in case of Andaman Islands (isolated populations) with longer ROH may have higher susceptibility to SARS-CoV-2. Apart from these known isolated populations, we have also found out several Dravidian speaking groups harbouring high homozygosity (Fig. 1). Interestingly, these Dravidian speakers with large size and numbers of homozygous segments are from both tribal as well as caste populations. Among the populations other than Dravidian, carrying homozygous segments of more than 150 Mb, only a single group, each of Himalayan region (Changpa) and Austroasiatic (Kissan) are present among studied populations. Thus, majority of larger segments were present among Dravidian speakers. Interestingly, in the analysed dataset we did not find any Indo-European speaking population carrying segments larger than 150 Mb. In most of the populations with the larger segments, it is pertinent that the smaller population size and high level of inbreeding have reduced the heterozygosity.

**Table 1:** The frequency distribution (95%CI) among Austroasiatic, Tibeto-Burman and Andamanese groups for the risk alleles observed in the present study.

Population group	*N*	rs10490770(C)	rs2285666(G)
Austroasiatic	106	0.302 (0.223–0.395)	0.274 (0.198–0.366)
Tibeto-Burman	96	0.063 (0.030–0.130)	0.135 (0.081–0.218)
Jarawa	19	0.263 (0.119–0.491)	0.579 (0.361–0.769)
Onge	17	0.294 (0.133–0.535)	0.353 (0.173–0.590)

In addition, with the studied populations, there are several isolated populations, e.g., language isolates-Nihali [64], genetic isolates-Abujhmaria [65], and many more who have shown the high ROH., Although, these populations are not well connected with the mainstream populations, however, there are high probabilities for them to contract with this virus seeing its nature of infectivity and range expansion. Furthermore, keeping in view of SARS-CoV-2 medical procedures, and lack of viable healthcare modern facilities, therefore, we suggest a high priority protection and utmost care for these isolated groups, so that we should not suffer to lose some of the living treasures of modern human evolution.

## Data Availability

All datasets generated for this study are included in the article.
